# Acquired Hemophilia A Presenting as a Spontaneous Soft Tissue Hematoma in an Elderly Male

**DOI:** 10.7759/cureus.84820

**Published:** 2025-05-26

**Authors:** John K Appiah, Chukwunonso B Ubanatu, Karna Desai, Richeal Asante, Haider Bin Khalid

**Affiliations:** 1 Internal Medicine, Geisinger Health System, Wilkes-Barre, USA

**Keywords:** acquired hemophilia a, autoimmune bleeding disorder, factor viii inhibitor, periprosthetic hematoma, spontaneous hematoma

## Abstract

Acquired hemophilia A (AHA) is a rare but potentially life-threatening autoimmune bleeding disorder characterized by autoantibodies against factor VIII. It often presents with spontaneous bleeding in patients without a personal or family history of coagulopathy, making diagnosis particularly challenging. We present a case of a 60-year-old male with a remote history of right total hip arthroplasty (THA) who developed a spontaneous intra-articular hematoma, initially mistaken for a periprosthetic infection. He later re-presented with expanding ecchymosis and anemia, ultimately diagnosed with AHA following hematology evaluation. This case highlights the diagnostic pitfalls and emphasizes the importance of considering acquired coagulopathies in unexplained bleeding.

## Introduction

Acquired hemophilia A (AHA) is a rare autoimmune bleeding disorder resulting from the development of autoantibodies that neutralize coagulation factor VIII. The incidence is estimated at 1.5 cases per million per year, making it a challenging diagnosis, particularly in patients without prior bleeding history [1]. AHA differs markedly from congenital hemophilia, primarily affecting older adults and often occurring in the context of autoimmune diseases, malignancy, postpartum state, or without an identifiable cause (idiopathic) [2,3].

Clinically, AHA is characterized by spontaneous bleeding into the skin, muscles, or soft tissues, rather than joints, which are more typical in congenital hemophilia. The diagnosis is usually suspected based on unexplained prolonged activated partial thromboplastin time (aPTT) with normal prothrombin time (PT) and confirmed by low factor VIII levels and the presence of inhibitors on mixing studies [4]. The rarity and nonspecific presentation often lead to delays in diagnosis and treatment, contributing to increased morbidity and mortality. Here, we describe a case of AHA in a patient presenting with symptoms initially attributed to orthopedic complications, illustrating the need for heightened clinical suspicion in atypical postoperative courses.

## Case presentation

A 60-year-old male with a history of right total hip arthroplasty (THA), performed nine years ago, presented to the emergency department with acute-onset right hip pain. He reported intermittent discomfort and pain in the hip over the preceding few days; the pain had significantly worsened to the point that he could no longer ambulate. He denied any trauma, recent falls, fever, chills, or anticoagulant use.

**Figure 1:**
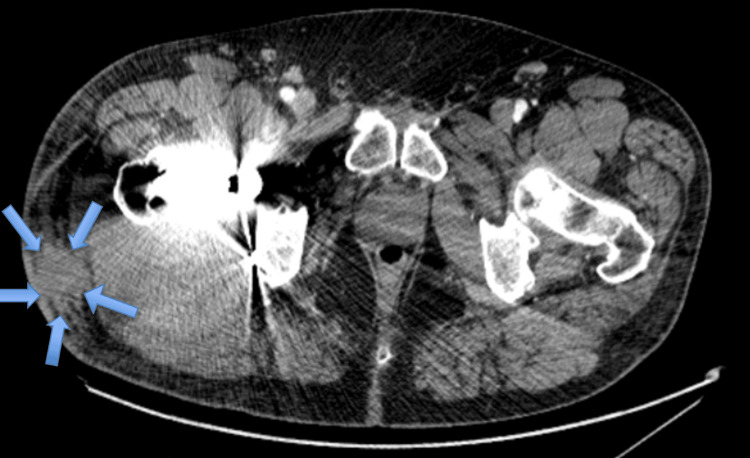
Axial CT showing a right lateral soft tissue hematoma adjacent to the hip arthroplasty. An axial CT image of the pelvis showing a right lateral soft tissue hematoma (blue arrows) adjacent to the total hip arthroplasty components. No pseudoaneurysm is identified.

On examination, he was afebrile and hemodynamically stable. The right hip was tender to palpation with severely restricted range of motion due to pain, but there was no erythema, swelling, or drainage. Initial laboratory evaluation revealed mild leukocytosis with elevated C-reactive protein (Table 1). A CT scan of the pelvis revealed degenerative lumbar changes and soft tissue hematoma adjacent to the prosthesis without vascular abnormality (Figure 1). These lab and imaging findings raised concerns about a possible periprosthetic infection.

**Table 1:** Initial laboratory evaluation on admission. Selected laboratory results, including complete blood count (CBC) and inflammatory markers. Notable findings include a mildly elevated white blood cell (WBC) count and increased C-reactive protein (CRP), suggestive of inflammation. All other parameters are within reference ranges.

Test	Result	Reference range
CBC: peripheral blood		
WBC (×10³/μL)	11.90	4.0-11.0
RBC (×10⁶/μL)	4.83	4.20-5.90
Hemoglobin (g/dL)	15.1	13.5-17.5
Inflammatory markers		
CRP (mg/L)	27	<10
ESR (mm/hour)	15	0-20

**Figure 2:**
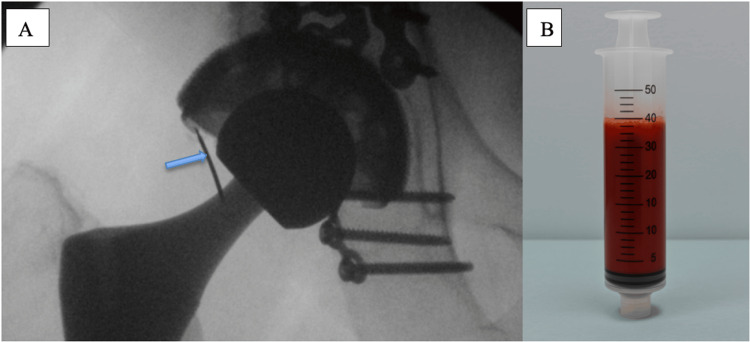
Fluoroscopic image of a right hip arthrogram with associated hemorrhagic joint aspiration. (A) Fluoroscopic image of the right hip demonstrating intra-articular contrast injection into a total hip arthroplasty. The blue arrow indicates the needle entering the joint space. (B) Syringe containing approximately 40 mL of grossly hemorrhagic fluid aspirated from the joint, consistent with hemarthrosis.

Ultrasound-guided aspiration of the right hip yielded approximately 40 cc of bloody, turbid fluid (Figure 2). Synovial fluid analysis is summarized in Table 2. Findings included a mildly elevated white blood cell (WBC) count with neutrophilic predominance and markedly increased red blood cells (RBCs), consistent with hemarthrosis. Synovial fluid cultures demonstrated no microbial growth. 

**Table 2:** Synovial fluid analysis from right hip aspiration. Synovial fluid obtained from ultrasound-guided aspiration of the right hip demonstrated mildly elevated WBCs with neutrophilic predominance and markedly elevated RBCs, consistent with hemarthrosis. No organisms were isolated in culture.

Parameter	Result	Reference range
Appearance	Red, turbid	Clear/colorless
Nucleated cells (cells/μL)	4,884	0-180 cells/uL
RBC count (cells/μL)	3,615,000	<2,000 cells/uL
Neutrophils (%)	82	<25
Lymphocytes (%)	12	0-78
Monocytes (%)	6	0-71

Despite aspiration, the patient’s symptoms did not improve, and his C-reactive protein (CRP) continued to rise. Given the lack of clinical progress and the equivocal nature of the aspiration, surgical irrigation and debridement were recommended. Coagulation studies at this stage, including PT and International Normalized Ratio (INR), were within normal limits. The patient was taken to the operating room, where a large, dark hematoma was discovered within the joint. There was no purulence or evidence of prosthetic loosening. The procedure was uncomplicated, and the patient was discharged on postoperative day 2.

**Figure 3:**
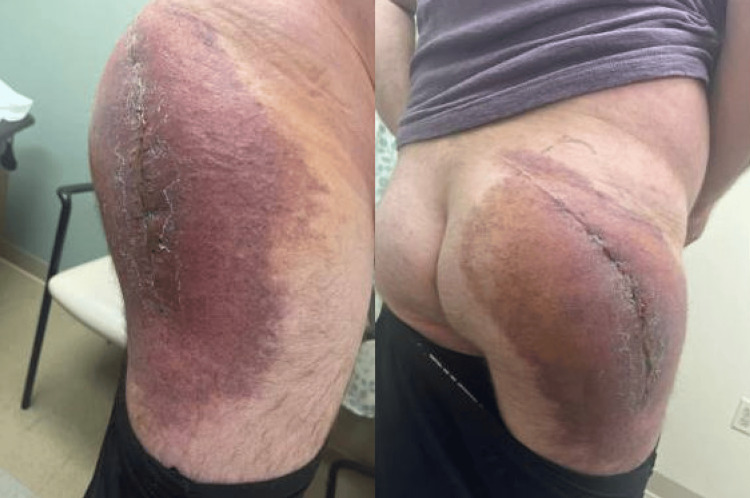
Extensive ecchymosis following incision and debridement of the right hip. Expanding right lateral hip ecchymosis noted at follow-up, raising concern for ongoing bleeding without history of trauma or anticoagulation.

At his four-week follow-up visit, the patient returned with new, rapidly expanding ecchymosis over the right lateral hip (Figure 3). Laboratory testing at this time revealed anemia, leukocytosis, and a prolonged aPTT. Coagulation and inhibitor studies were obtained and are summarized in Table 3. He was found to have a factor VIII level <1%, and mixing studies confirmed the presence of an inhibitor, consistent with AHA. 

**Table 3:** Key laboratory findings supporting diagnosis of acquired hemophilia A. Selected laboratory results highlighting a prolonged activated partial thromboplastin time (aPTT), critically low factor VIII activity, and a positive mixing study consistent with an acquired factor VIII inhibitor. Normal haptoglobin and elevated reticulocyte percentage support bleeding rather than hemolysis as the cause of anemia.

Parameter	Result	Reference Range	Interpretation
Hemoglobin (g/dL)	8.4	13.5-17.5	Low: consistent with anemia
INR	1.1	0.8-1.2	Normal
aPTT (seconds)	51	21-38	High: suggests coagulopathy
Factor VIII activity (%)	1	50-150	Critically low: indicates severe deficiency
Mixing study	Rosner Index: 26.42 (H); aPTT: 53 → 39 (immediate), 48 (60 min)	Rosner <15%; aPTT 21-38 sec	Incomplete correction and elevated Rosner index, consistent with factor VIII inhibitor
Factor VII activity (%)	105	60-150	Normal: helps exclude broader factor deficiency
Reticulocyte(%)	8.83	0.80-1.90	Elevated: suggests increased marrow response to anemia
Haptoglobin (mg/dL)	42	30-200	Normal: no evidence of hemolysis
Lupus anticoagulant	Not detected	Negative	No evidence of lupus anticoagulant by clot-based methods

**Figure 4:**
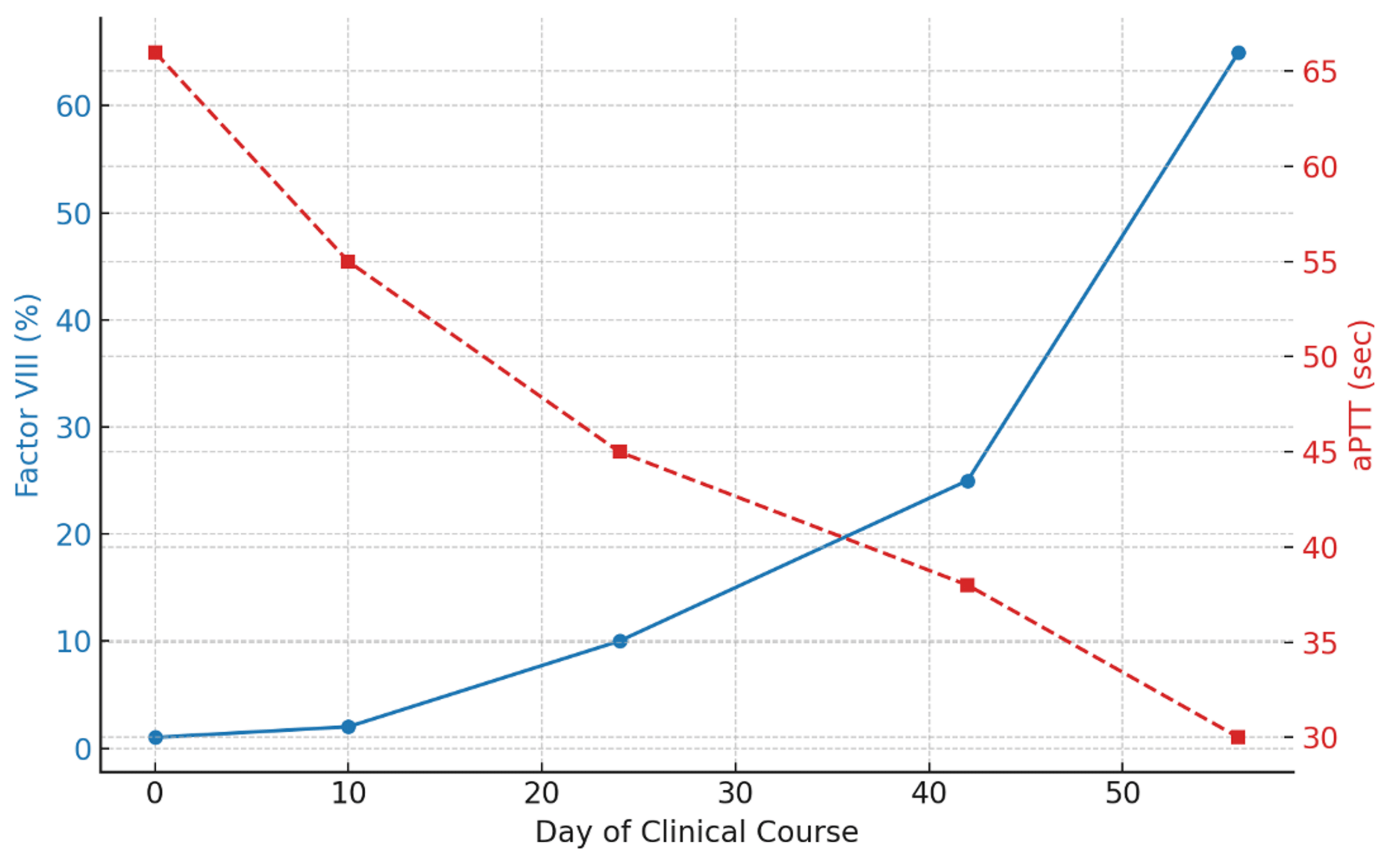
Recovery of coagulation parameters during the clinical course. Serial laboratory values demonstrating a progressive increase in factor VIII activity (%) and concurrent normalization of activated partial thromboplastin time (aPTT) during the patient’s clinical course, reflecting effective treatment of acquired hemophilia A.

The patient was readmitted and started on recombinant activated factor VII, corticosteroids, and rituximab. He completed four cycles of rituximab and underwent a prolonged steroid taper. His symptoms gradually improved, and his factor VIII levels rose above 5% before discharge. Figure 4 demonstrates recovery of coagulation function during the clinical course, evidenced by rising factor VIII levels and normalization of aPTT with treatment.

## Discussion

AHA is a rare but potentially fatal disorder that must be distinguished from more common causes of bleeding. While inherited bleeding disorders usually manifest in childhood, AHA typically arises in older adults with no personal or family history of coagulopathy [1,5]. It can be idiopathic or associated with underlying conditions such as autoimmune diseases, malignancies, dermatologic disorders, or even drug exposure [6,7]. In about 50% of cases, no clear etiology is found [2,3].

Our patient’s presentation initially mimicked a postoperative complication, hematoma versus infection, in a patient with a prior hip arthroplasty. His normal initial coagulation panel (without aPTT) and mildly elevated inflammatory markers made infection seem plausible. However, recurrence of bleeding, expanding hematoma, and progressive anemia in the absence of anticoagulation or trauma were red flags.

In retrospect, the absence of an aPTT before surgical intervention represents a key diagnostic gap. Given the clinical context of a painful, aspirate-positive joint and elevated inflammatory markers, the working diagnosis was periprosthetic infection. With PT and INR within normal limits and no bleeding history, aPTT was not routinely pursued. This case underscores the importance of expanding the coagulation evaluation in atypical presentations, especially when standard interventions fail to produce clinical improvement.

The diagnosis of AHA depends on three critical findings: isolated prolonged aPTT, low factor VIII activity, and failure to correct in mixing studies [8]. Additional confirmatory steps include the Bethesda assay to quantify inhibitor titers and ruling out lupus anticoagulant. In our case, a lupus-sensitive aPTT and normal DRVVT ratio helped confirm the diagnosis. Treatment includes hemostatic therapy (e.g., bypassing agents like recombinant activated factor VII) and immunosuppressive therapy, typically corticosteroids with or without rituximab or cyclophosphamide [9,10]. Our patient responded well to a combination of rituximab and steroids, with normalization of factor VIII levels over several weeks.

AHA should be suspected in any patient with unexplained bleeding and isolated aPTT elevation, especially if standard interventions fail or bleeding is recurrent. Early involvement of hematology is essential to guide immunosuppressive and hemostatic management.

## Conclusions

AHA, though rare, must be considered in the differential for spontaneous hematoma, especially in elderly patients with no bleeding history or anticoagulation use. Delays in diagnosis can lead to significant morbidity or mortality. This case emphasizes the value of maintaining a broad differential when managing postoperative complications and recognizing when the clinical course deviates from expected surgical outcomes. Furthermore, early recognition and multidisciplinary collaboration, particularly involving hematology, are essential for timely diagnosis, appropriate factor replacement, and immunosuppressive therapy. Continued awareness and education about this rare disorder may reduce diagnostic delays and improve patient outcomes.
